# Repurposing Antidiabetic Drugs for Gangrene: A Mendelian Randomization and Text Mining Study

**DOI:** 10.7150/ijms.111050

**Published:** 2025-06-12

**Authors:** Chenfeng Wang, Huiwei Wang, Ting Feng, Yihe Hu, Feng Liang

**Affiliations:** 1 Department of Orthopedic Surgery, The First Affiliated Hospital, College of Medicine, Zhejiang University, Hangzhou, Zhejiang 310030, China.; 2 Department of Dermatology and Venerology, First Hospital of Jilin University, Changchun, Jilin 130021, China.; 3 Department of Dermatology, The University of Hong Kong-Shenzhen Hospital, Haiyuan 1st Road, Futian District, Shenzhen, Guangdong 518053, China.

**Keywords:** Gangrene, Mendelian randomization, Diabetes, Drug discovery, Type 1 Diabetes Mellitus, Type 2 Diabetes Mellitus, Genome-wide association studies (GWAS), Protein-protein interaction (PPI)

## Abstract

**Objective:** Gangrene has been a problem for many people with diabetes. Besides, the relationship and pathomechanism of diabetes-induced gangrene (DG) are still unclear. The aim of this study was to investigate the causal relationship between diabetes and gangrene through Mendelian randomization (MR) and to identify potential therapeutic agents using bioinformatics analysis.

**Method:** Summary data from genome-wide association studies (GWAS) were utilized to evaluate the connection between two types of diabetes and gangrene risk using a two-sample MR design. Single nucleotide polymorphisms (SNPs) that were significantly associated with diabetes were selected as instrumental variables, and their validity was verified by F-statistics and other methods. Next, we used text mining and protein-protein interaction (PPI) networks to filtrate significant genes for drug-gene interaction (DGI) to identify prospective medications for the therapy of DG.

**Results:** Through multiple methods analysis (IVW, MR-Egger and MR-PRESSO etc.), MR analysis showed that genetic susceptibility to type 1 diabetes was related to a higher risk of gangrene risk (OR: 1.19, 95% CI: 1.04-1.36, P-value: 0.0134), while type 2 diabetes mellitus (T2DM) could also increase the gangrene risk (OR: 1.57, 95% CI: 1.05-2.33, P-value: 0.0269). The outcomes of text mining disclosed 50 genes enriched in NOD-like receptor and RAGE signaling pathways commonly associated with both diabetes and gangrene for PPI analysis. Subsequent DGI analysis revealed six genes targeted by 12 drugs (DGI score > 5), presenting them as candidates for treating DG.

**Conclusion:** In conclusion, this study not only validates the causal effect of diabetes on gangrene risk but also identifies several potential therapeutic agents (CILAZAPRIL, RESATORVID, SILTUXIMAB, and OLOKIZUMAB) by integrating bioinformatics analysis, providing new directions for future clinical interventions.

## Introduction

Diabetes mellitus, an intricate metabolic disorder characterized by persistent hyperglycemia, constitutes a formidable global health challenge with a burgeoning prevalence on a worldwide scale^1^. Gangrene, an austere manifestation of vascular complications in diabetes, emanates from compromised blood supply to tissues, precipitating tissue necrosis^2^. Ischemia, impaired angiogenesis, and heightened susceptibility to infections collectively contribute to the initiation and progression of gangrene in individuals with diabetes^3^. The prevailing therapeutic paradigm for gangrene in diabetes heavily relies on conventional interventions, encompassing revascularization procedures and wound care^4^. Nonetheless, these modalities frequently prove insufficient in arresting the advancement of gangrene or achieving comprehensive resolution, thereby engendering a heightened incidence of recurrent infections and amputations^5^. The circumscribed success of extant treatments accentuates the imperative necessity for pioneering strategies to redress the unmet medical exigencies in the management of gangrene in diabetes.

Diabetes can also be divided into type 1 and type 2 diabetes mellitus (T1DM and T2DM), collectively affecting 537 million adults worldwide in 2021. Among these, T2DM is the most prevalent, representing 90% of diabetes cases globally^6^. Both forms of diabetes are associated with significant microvascular and macrovascular complications. Diabetic foot, a severe microvascular complication of diabetes mellitus, carries a lifetime risk of 15-25% for progression to diabetic foot ulcer (DFU) among affected individuals, with 20-30% of these cases advancing to gangrene^7^. Epidemiological studies indicate this condition exhibits a higher prevalence in patients with T2DM compared to their T1DM counterparts^8^, T1DM patients exhibit a comparable or even higher age-adjusted risk, particularly among those with long disease duration and poor glycemic control. This discrepancy reflects distinct underlying pathophysiological mechanisms: in T1DM, gangrene development is primarily driven by autoimmune-mediated endothelial dysfunction and accelerated atherosclerosis, whereas complications in T2DM are often exacerbated by metabolic syndrome components, including obesity and dyslipidemia^9^. Nevertheless, hyperglycemia-induced oxidative stress and impaired angiogenesis serve as shared pathological pathways in both T1DM and T2DM, highlighting potential overlaps in therapeutic targets^10^.

Antidiabetic medications, encompassing various classes such as insulin, sulfonylureas, metformin, thiazolidinediones, glucagon-like peptide-1 (GLP-1) receptor agonists, and dipeptidyl peptidase-4 (DPP-4) inhibitors, have played a pivotal role in diabetes management by targeting diverse aspects of glucose homeostasis^11^. Beyond their primary role in glycemic control, these medications exhibit varied effects on inflammation, endothelial function, and cellular processes, suggesting potential benefits beyond their established functions^12^. The repurposing of antidiabetic drugs for the treatment of gangrene holds promise for several reasons. Firstly, the well-established safety profiles of these medications, extensively utilized in diabetes management, provide a solid foundation^13^. Repurposing presents a practical approach to capitalize on these existing drugs, potentially accelerating the translation of research findings into clinical applications^14^. Secondly, the pleiotropic effects of antidiabetic drugs on vascular function, inflammation, and tissue repair position them as attractive candidates for addressing the multifaceted pathogenesis of gangrene^15^. Repurposing existing pharmacotherapies with established safety profiles offers a strategic advantage. It can expedite the translation of research findings into clinical practice.

The continuous advancement of algorithmic analysis techniques has ushered in novel research methodologies for the investigation of correlations among diverse medical conditions. This avenue holds significant promise as it provides a means to elucidate the intricate mechanisms and potential therapeutic approaches for gangrene. In recent years, the Mendelian randomization (MR) method has emerged as a robust solution to address these methodological challenges. Traditional observational studies are often disturbed by confounding factors and reverse causality, but MR method can effectively avoid these biases by using genetic tool variables, so as to provide more reliable causal inference. MR, grounded in genetic epidemiology, leverages instrumental variables (IVs) to explore causality while mitigating the impact of confounding factors. It is specifically tailored to counteract biases stemming from reverse causation and unmeasured confounding. MR is an innovative tool in genetic epidemiology to explore causal associations through instrumental variables, effectively reducing reverse causality and unmeasured confounding bias. Combined with bioinformatics analysis, we can deeply reveal the mechanism of gene and molecular action in the occurrence of disease, and provide new theoretical guidance for the treatment of diabetic gangrene.

**Figure 1:**
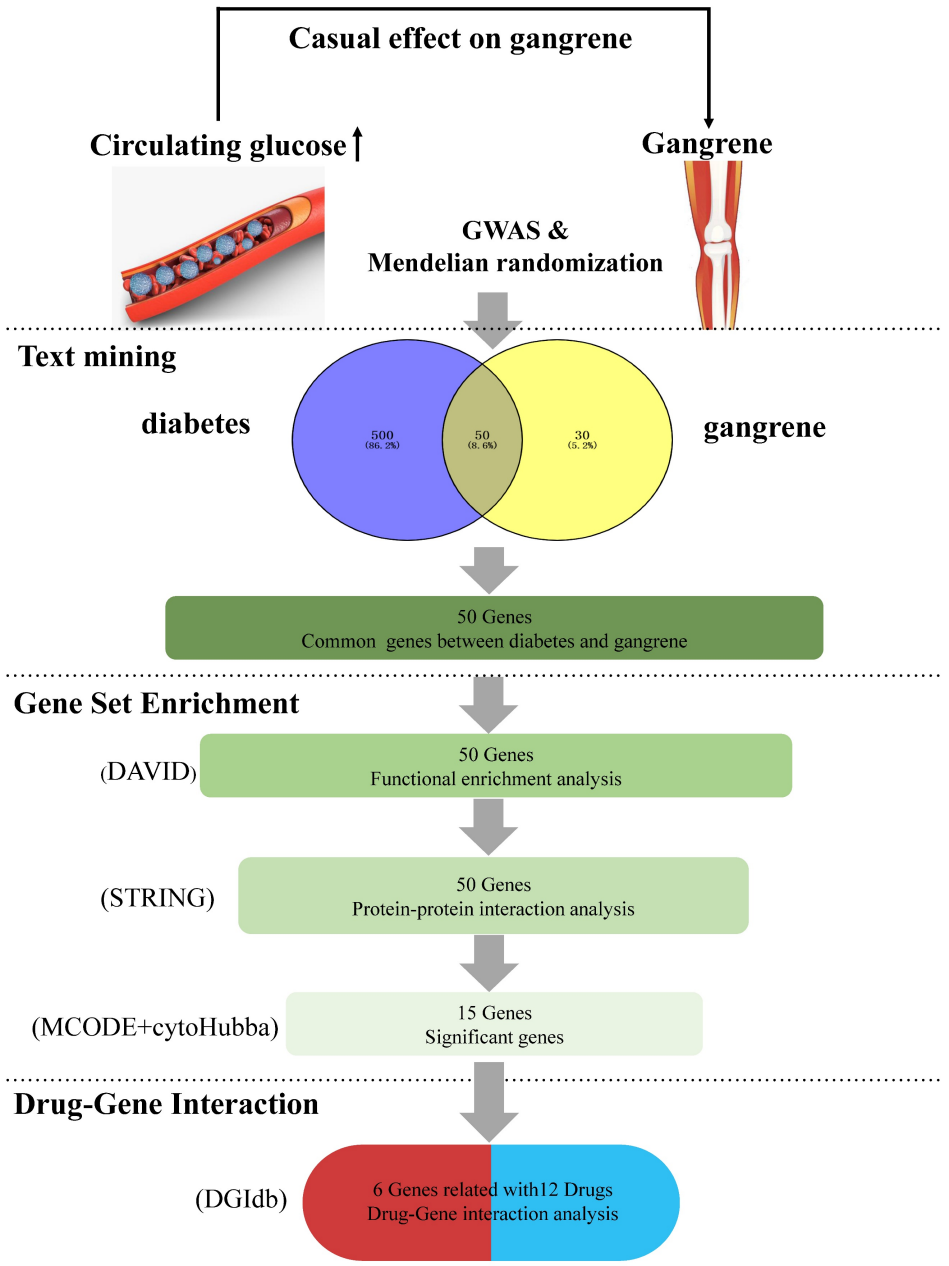
** A comprehensive summary of the overall results obtained from data mining.** (1) Two-sample Mendelian randomization analyses between two types of diabetes and gangrene. (2) Intersecting genes: the process of obtaining intersecting genes involved the utilization of specific search terms, 'diabetes' and 'gangrene' in GENCLIP3. This yielded 550 genes for diabetes and 80 genes for gangrene, ultimately leading to the identification of 50 common genes shared between the two conditions. (3) Functional annotations: gene functional enrichment analysis was performed using the DAVID tool. Subsequently, 15 hub symbols were identified using STRING and Cytoscape software. (4) Drug-gene interaction analysis: 12 potential medicines were acquired by DGIdb.

This study aims to systematically explore the pathogenesis of diabetes-induced gangrene through MR Analysis and bioinformatics methods, identify potential therapeutic targets and drug candidates, and provide a scientific basis for precision medicine, as illustrated in Figure 1.

## Methods

### Study protocol

To demonstrate the impact of diabetes on gangrene, we employed a two-sample MR design (hospital discharge or cause of death: we selected the study population based on ICD-10 code R02 (gangrene) and excluded signs and symptoms related to the circulatory and respiratory systems to ensure disease specificity of the case population. Initially, we identified the gene(s) encoding the target protein(s) associated with each antidiabetic drug class through data obtained from Drugbank and ChEMBL databases. Next, we used MR analysis to merge two datasets and evaluate the impact of genetic diversity in diabetes on the probability of gangrene. Supplementary Table 1 lists all of the GWAS datasets involved, including genetic variations for diabetes and gangrene, detailing the sample size, study design, and associated characteristics of each dataset. We followed the STROBE-MR guidelines outlined in the STROBE-MR Supplement.

### Genetic data processing

We used the R package 'TwoSampleMR' to process summary statistics for exposures in order to guarantee the identification of legitimate MR equipment. It is noteworthy that correlated instruments, particularly those in linkage disequilibrium, have the potential to add bias into measures of mean square error. To address this issue, we clumped the exposure SNPs (r^2^ = 0.001, window = 10 Mb, P-value threshold =5×10⁻⁸) with reference to the 1000 Genomes European dataset, which is to ensure independence between the selected SNPs while retaining a sufficient number of valid instrumental variables. Following this step, the effect alleles of the exposure and outcome datasets were aligned onto a single reference strand in order to undergo harmonization. In processing the data, we paid particular attention to the removal of linkage unbalanced SNPs to reduce the bias introduced by these SNPs. In addition, all potential instrumental variables were tested for heterogeneity to ensure their suitability for MR Analysis. At data alignment, the effect alleles of all exposure and outcome datasets are aligned to the same reference chain. In the wrong direction of the reference chain, we have corrected it to ensure the consistency and accuracy of the data. For SNPs that cannot be explicitly referenced, we excluded them to avoid interfering with the analysis results.

### MR analyses

To evaluate causality, we conducted a thorough MR analysis using multiple methods including random-effect or fixed-effect inverse-variance weighted (IVW), maximum likelihood, MR-Egger, weighted median, weighted mode, and MR-PRESSO. The IVW method is suitable for situations without significant heterogeneity, while the MR-Egger method can adjust the potential bias caused by gene pleiotropy. The weighted median method is robust to the inefficiency of a few instrumental variables, while the MR-PRESSO method is used to detect and correct the bias of instrumental variables.

We primarily relied on the conventional IVW method but also considered heterogeneity through Cochran's Q analysis. We used the fixed-effects IVW technique as our primary analytical strategy if P-values were above 0.05 and there was no indication of heterogeneity. The study utilized the random-effects IVW approach in cases of substantial heterogeneity (P < 0.05). Maximum likelihood, MR-Egger, weighted median, weighted mode, and MR-PRESSO as supplements to the IVW method. The results were presented as odds ratios with 95% confidence intervals, and P < 0.05 was used to indicate statistical significance. We require consistency in the directionality of results between different methods. If the IVW method shows significance and the other methods do not, but the results are in the same direction, it is considered a positive result. We used Cochran's Q test and I² statistics to assess the heterogeneity between the methods. The results of the MR analysis were visualized by scatter plots. The use of these various methods allowed us to thoroughly explore causality.

### Sensitivity analysis

To ensure the reliability of causal inference, we combined a variety of methods for sensitivity analysis. Cochran's Q test was used to examine the heterogeneity of SNP effects. If there was significant heterogeneity (P < 0.05), the random-effects IVW model was used. The MR-PRESSO and MR-Egger methods were used to evaluate and correct possible pleiotropic biases. MR Steiger's test helps confirm causal directionality.

### Text mining

The GenCLiP3 website (http://ci.smu.edu.cn/genclip3/analysis.php) provided gene sets associated to diabetes and gangrene. "All human genes", "Search in MEDLINE" and keywords (gangrene and diabetes) were typed into the website to obtain the two gene lists.

### Gene ontology (GO) and Kyoto Encyclopedia of Genes and Genomes (KEGG) analysis

The DAVID (http://www.david.com) was used to annotate genes representing gangrene and diabetes, as well as biological processes (BP), cellular components (CC), and molecular pathways. An important part of the functional annotation process was identifying the key symbols related to the pathology of gangrene by using KEGG analysis.

### Protein-protein interaction (PPI) network

The common symbols were entered into the confidence (score 0.400) STRING database (http://string-db.org). A complex procedure was carried out to separate hub genes using the Cytoscape program, which made use of the Molecular Complex Detection (MCODE) and cytoHubba apps. The cytoHubba cutoff was carefully set at the highest point, capturing the top 15 genes within its narrow scope, whilst the MCODE cutoff parameters remained as they were.

### Drug-gene interaction (DGI)

The group of interacting genes that resulted from the combination of MCODE and cytoHubba apps served as the analytical foundation for further Drug-Gene Interaction (DGI) analysis. The stringent criteria established a high bar, meaning that a DGI score had to rise to a minimum of 5, while at the same time a classification with absolute accuracy and clear categorization had to be made. Such strict standards result in a detailed and careful assessment, providing a higher degree of selectivity when it comes to identifying the complex interactions between genetic entities and pharmacological modalities.

## Results

### MR and sensitivity analysis

**Figure 2:**
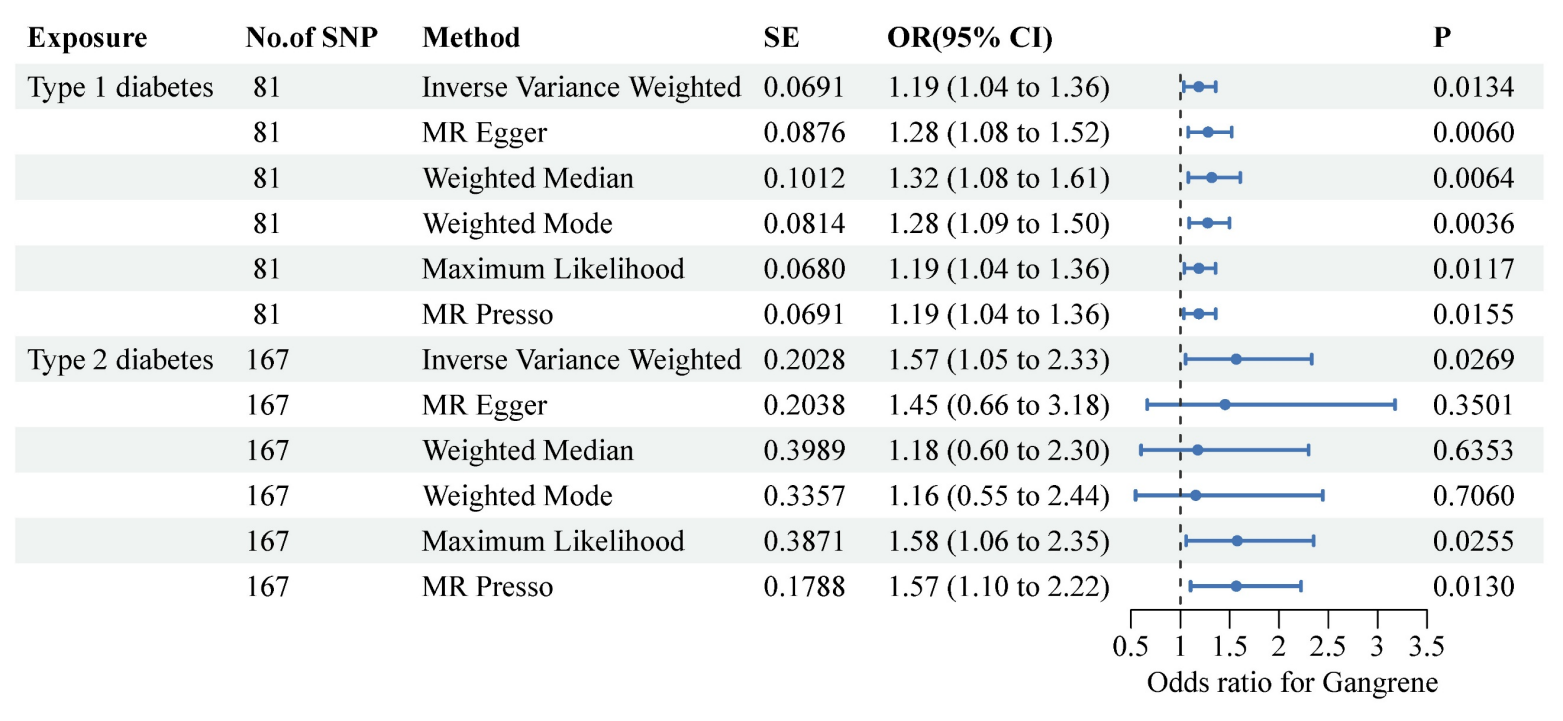
** Forest plots present the associations between single-nucleotide polymorphisms and both diabetes and the risk of gangrene.** Two-sample Mendelian randomization analyses between two types of diabetes and the risk of gangrene were performed. Each type of diabetes is depicted on the X-axis, while the estimated effect is shown on the Y-axis. Filled point estimates represent statistically significant results with a P-value less than 0.05. The results are interpreted as odds ratios with corresponding 95% confidence intervals for gangrene risk per each standardized increment. MR, Mendelian randomization.

To ensure the validity of the instrumental variables, we calculated the average F statistic for each type of diabetes. An F statistic of less than 10 usually indicates potential instrumental variable bias. For both T1DM and T2DM, we found that the F statistic was greater than 10, indicating that our instrumental variable is valid. The IVW method demonstrated a positive correlation with the type 1 diabetes mellitus (T1DM) (OR: 1.19, 95% CI: 1.04-1.36, P-value: 0.0134) and type 2 diabetes mellitus (T2DM) (OR: 1.57, 95% CI: 1.05-2.33, P-value: 0.0269) (Figure 2), clearly showing that T1DM and T2DM were significantly associated with the risk of gangrene. In the MR analyses, we examined both vertical and horizontal pleiotropy. We then conducted the MR-Egger test for horizontal pleiotropy, which yielded no evidence of such pleiotropy in T1DM and T2DM (P>0.05 for all), suggesting that there was no bias in our MR estimates (Supplementary Table 2). Our leave-one-out analyses further validated the robustness of our findings.

### Common symbols

Following a thorough investigation utilizing text mining methods, we discovered 550 distinct genes connected to diabetes and an additional 80 distinct genes connected to gangrene specifically. Of these, it was discovered that a subset of exactly 50 genes were shared by gangrene and diabetes. This genetic junction is the central focus of our follow-up study, providing a critical basis for a thorough examination of the same molecular mechanisms underlying the pathophysiological expression of diabetes and the beginning of gangrene.

### Functional annotations

The quintet of paramount biological processes (BP), each characterized by a discerning false discovery rate (FDR), is sequentially articulated as follows: (1) response to external stimulus (FDR=2.13E-17), (2) response to other organism (FDR=2.66E-11), (3) response to external biotic stimulus (FDR=2.66E-11), (4) negative regulation of multicellular organismal process (FDR=2.66E-11), and (5) response to biotic stimulus (FDR=4.14E-11). Within the ambit of cellular components (CC), the superlative stratum encompasses (1) extracellular space (FDR=2.7E-18), (2) extracellular region (FDR=2.44E-12), (3) extracellular region part (FDR=3.24E-12), (4) cytoplasmic vesicle lumen (FDR=9.39E-06), and (5) vesicle lumen (FDR=9.39E-06). In the purview of molecular functions, the preeminent roles were embodied by (1) receptor binding (FDR=6.49E-10), (2) hormone activity (FDR=2.35E-04), (3) cytokine activity (FDR=3.32E-04), (4) identical protein binding (FDR=0.0089), and (5) G-protein coupled receptor binding (FDR=0.0089) were the top 5.

**Figure 3:**
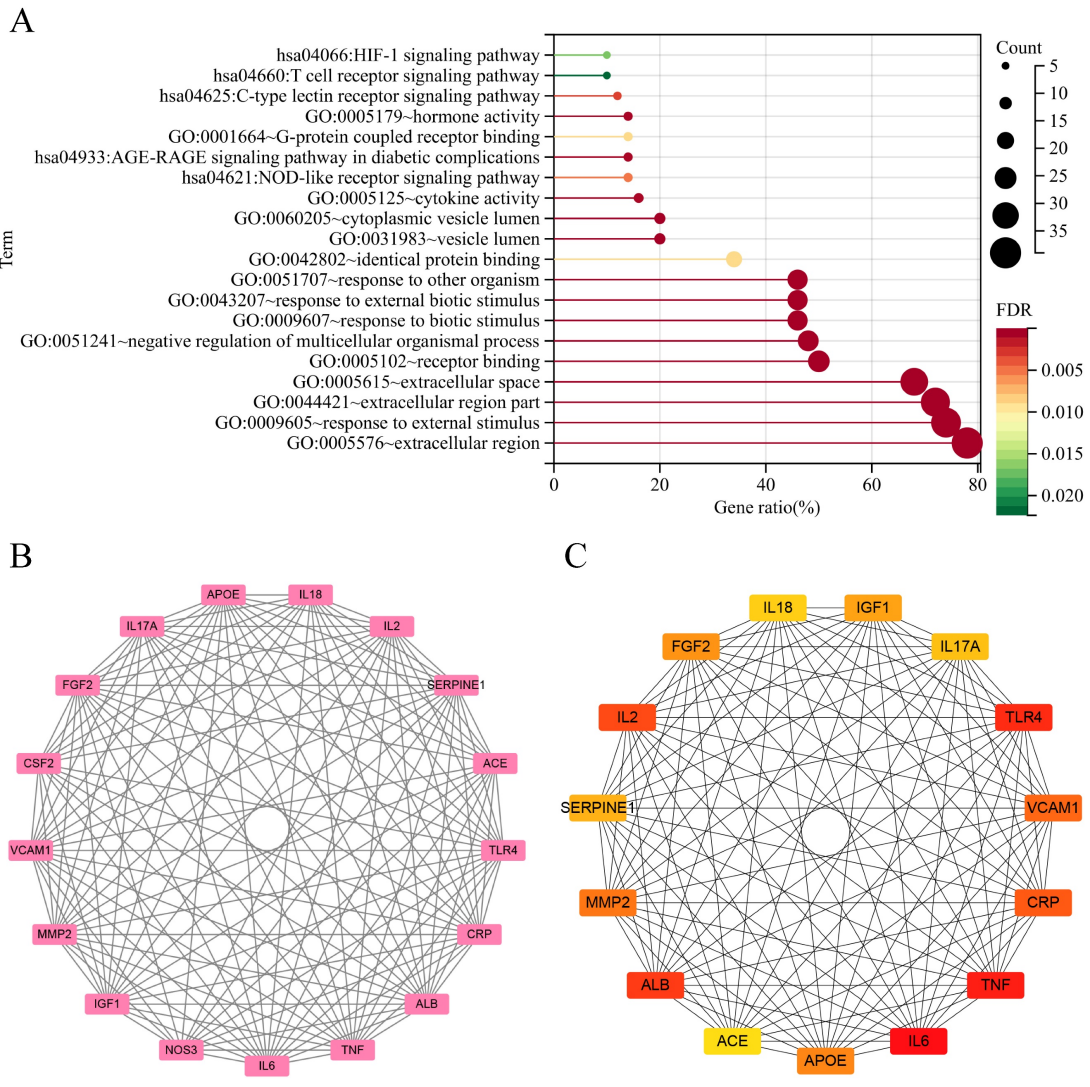
** Functional annotations and protein network analysis.** (A)With dots of varying size and color denoted the number of genes involved and their reliability, respectively. (B) The most significant cluster: There are 17 edges and 132 nodes. (C) CytoHubba analysis: The top 15 genes were analyzed.

KEGG pathway studies demonstrated the participation of (1) AGE-RAGE signaling pathway in diabetic complications (FDR=3.36E-04), (2) C-type lectin receptor signaling pathway (FDR=0.0028), (3) NOD-like receptor signaling pathway (FDR=0.0048), (4) HIF-1 signaling pathway (FDR=0.0171), and (5) T cell receptor signaling pathway (FDR=0.0224) (Figure 3A).

### PPI network and key symbols acquirement

In the PPI network constructed by interacting 50 symbols, a clear module consisting of 17 genes was identified by cluster analysis using the MCODE technique, as shown in Figure 3B. Meanwhile, the use of cytoHubba algorithms—which are intended to identify major nodes in biological networks—made it easier to extract the top 15 symbols, as shown in Figure 3C.

### Potential therapeutics

**Figure 4:**
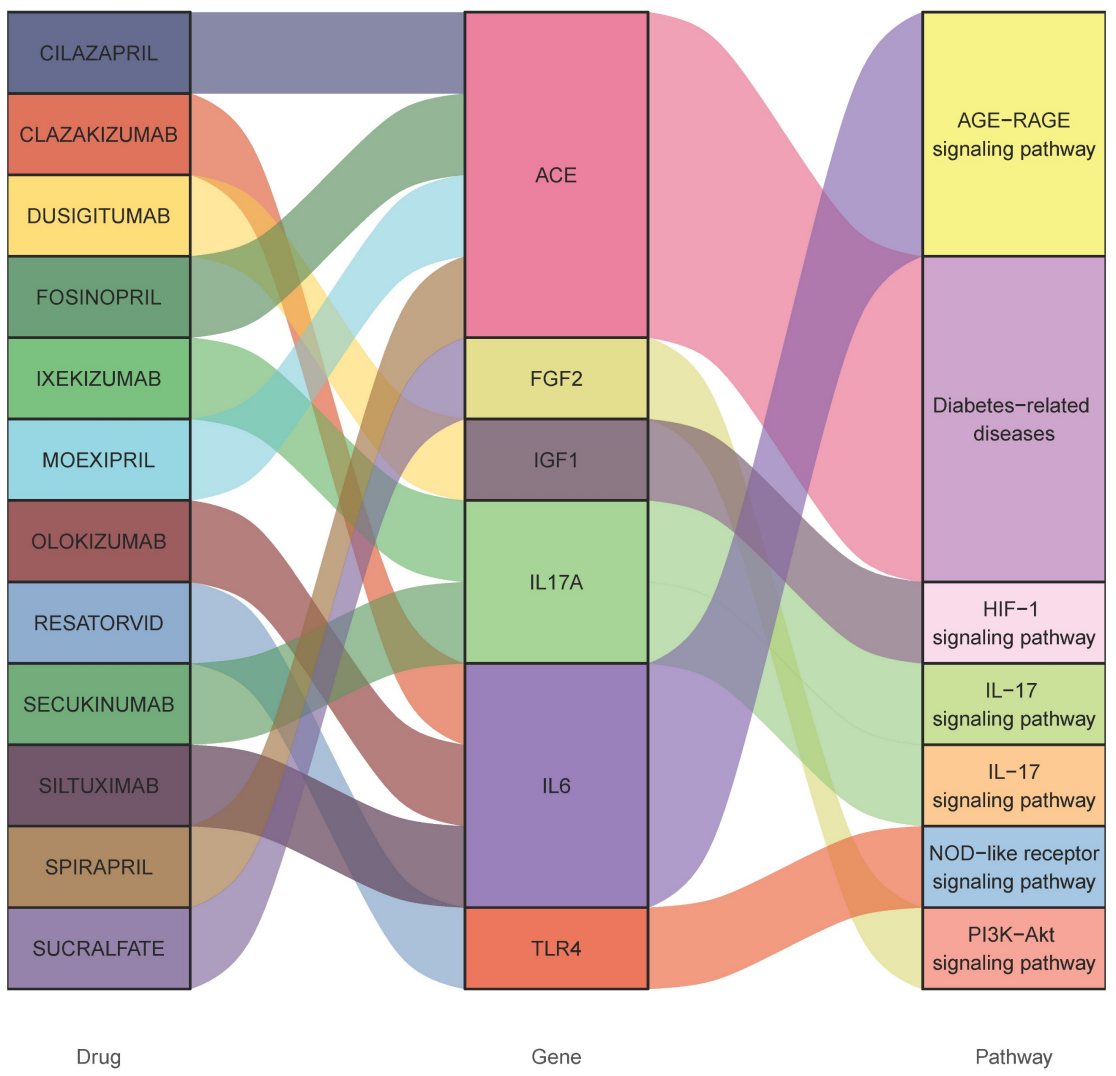
** Interactions between drug-gene-pathway.** The diagram provides insights into the interplay between 6 genes targeting 12 drugs and 5 pathways, providing an insightful visualization of the intricate relationships involved.

Consequently, 12 drugs (OLOKIZUMAB, DUSIGITUMAB. RESATORVID, SECUKINUMAB, SILTUXIMAB, SPIRAPRIL, FOSINOPRIL, CLAZAKIZUMAB, CILAZAPRIL, IXEKIZUMAB, MOEXIPRIL, SUCRALFATE) corresponding to 6 essential genes (IL17A- interleukin 17 alpha, FGF2- fibroblast growth factor 2, ACE- angiotensin-converting enzyme, IGF1- insulin like growth factor 1 and TLR4- Toll like receptor 4, IL6-interleukin 6) were discovered to influence gangrene (Figure 4 and Table 1).

## Discussion

This work is the first attempt to explore whether diabetes was associated with gangrene risk, which suggested that there was a casual effect of diabetes on the gangrene. In addition to this primary investigative focus, the secondary aim of this study encompasses the identification of key genes, the discernment of pharmacologically targetable therapeutic agents, and the elucidation of underlying pathogenic mechanisms.

The gene SLC5A2, which is implicated in both diabetes and gangrene, serves as the specific target for SGLT2 inhibitors in diabetic therapy. Beyond their blood glucose-lowering effects, SGLT2 inhibitors exhibit pleiotropic properties, influencing crucial non-glycemic pathways. This results in end-organ protection, as evidenced by their renoprotective effects in diabetic kidney disease. Moreover, these inhibitors can directly target podocytes, depending on the maintenance of actin cytoskeleton architecture^16^. Hence, SGLT2 inhibitors emerge as potential candidates for gangrene therapy.

As a result, six pathways were filtered and six hub genes linked with twelve medications. Skeletal development is significantly impacted by FGF2, which is generated by mesenchymal and epithelial cells^17^. However, it has been claimed that aluminum foil mixed with basic fibroblast growth factor (bFGF) may be used to treat gangrene^18^ and a high-glucose environment causes its expression to decrease, which results in cell proliferation disorder^19^. Moving on to IL17A, a proinflammatory cytokine, it is secreted by cells as a result of diabetes, and this can result in a number of issues^20^. Furthermore, this study examined IL6, an inflammatory cytokine implicated in the pathogenesis of diabetes as well as stimulating an inflammatory response^21^.

ACE inhibitors find widespread use in treating cardiovascular diseases and diabetes, significantly decreasing the incidence of various complications^22^, ^23^. Insulin and IGF1 signaling resistance in β cells contributes to the pathogenesis of diabetes, with lower genetically influenced IGF1 associated with a higher diabetes risk^24^. Therefore, activating IGF1 emerges as a potential target for diabetes management. TLR4, a pattern recognition receptor, becomes a focal point for intervention. In T2DM, reducing TLR4-mediated inhibition of the TLR4-nuclear factor kappa B (NF-κB) pathway lowers the inflammatory response and related problems^25^.

As mentioned above, the expressions of IL17A, ACE, TLR4, and IL6 are up-regulated with diabetes that promote the progression of gangrene, whereas the expressions of FGF2 and IGF1 are down-regulated. Therefore, this study only addresses inhibitors of up-regulated genes. Based on the DGI scores, the top 4 potential drugs are CILAZAPRIL (ACE inhibitor, 24.73), RESATORVID (TLR4 inhibitor, 11.24), SILTUXIMAB (IL6 inhibitor, 9.89) and OLOKIZUMAB (IL6 inhibitor, 9.89). CILAZAPRIL is primarily employed in the treatment of gastric cancer, ulcerative colitis, hypertension, and congestive heart failure. It serves to mitigate organ damage resulting from hyperglycemia^26^, ^27^. RESATORVID, functioning as a TLR4 inhibitor, exhibits the capacity to ameliorate osteoarthritis pathology by impeding chondrocyte pyroptosis and degeneration. Additionally, it mitigates ROS-induced macrophage inflammation through the TLR4/MyD88/NF-κB/NLRP3 pathway^28^. Furthermore, a reduction in TLR4 expression proves beneficial in alleviating diabetic-induced vascular complications and diminishing reactive oxygen species (ROS) production induced by hyperglycemia^29^. As IL6 inhibitors, SILTUXIMAB and OLOKIZUMAB are now being investigated for Castleman disease^30^ and rheumatoid arthritis^31^.

DG, characterized by tissue ischemia, necrosis, and secondary bacterial infection, considers the endocrine system to be a vital intermediary. Advanced glycation end products (AGEs) have a major effect on the metabolism and function of endothelial cells through their interaction with the receptor for AGEs (RAGE). This interaction induces endothelial cell pyroptosis through the HIF-α-RAGE-NLRP3 pathway^32^. The NOD-like receptor signaling pathway plays a role in the development of Type 2 Diabetes Mellitus (T2DM) by mediating inflammatory death in pancreatic β-cells^33^. As for the diabetes, the impaired PI3K-Akt pathway exacerbates the pathogenic cascade, further predisposing to gangrene4^34^.

The 12 candidate drugs identified in this study—including antioxidants, anti-inflammatory agents, and angiogenesis modulators—are likely to target mechanisms common to both T1DM- and T2DM-associated gangrene, such as chronic inflammation and vascular dysfunction. Notably, metformin, although primarily prescribed for T2DM, has demonstrated pleiotropic effects on endothelial repair in preclinical models of T1DM^35^. Conversely, immunomodulators such as anakinra may hold particular therapeutic potential for T1DM due to their ability to mitigate autoimmune-driven vascular injury^36^. Nevertheless, the differential predominance of insulin resistance in T2DM versus absolute insulin deficiency in T1DM may necessitate subtype-specific dosing strategies or combination therapies. Importantly, current clinical trials evaluating gangrene therapies seldom stratify outcomes based on diabetes subtype, resulting in a notable evidence gap^37^. Future validation studies should explicitly compare pharmacokinetic profiles and therapeutic windows between T1DM and T2DM cohorts to refine precision medicine approaches and enhance clinical efficacy.

The extant body of literature has extensively examined the cardiovascular advantages conferred by antidiabetic drugs. In a departure from previous research, the present study uniquely contributes to the field by elucidating the potential of these drugs in preventing gangrene. Employing Mendelian randomization methodology imparts a heightened level of rigor to the findings, establishing a causal link and fortifying confidence in the identified associations. The discernment of specific drug classes correlated with a diminished risk of gangrene imparts actionable insights for both clinical practitioners and researchers. Beyond its immediate findings, this study not only lays the groundwork for future research initiatives but also underscores the imperative of validation across diverse demographic cohorts and healthcare environments. The incorporation of real-world evidence, encompassing data derived from electronic health records and pragmatic clinical trials, offers supplementary insights into the efficacy and safety of repurposed antidiabetic drugs within routine clinical practice^11^. The investigation into the mechanistic pathways through which antidiabetic drugs manifest their protective effects on gangrene assumes pivotal significance. Systematic scrutiny at the cellular and molecular levels promises to reveal specific targets and pathways, thereby facilitating the development of precision-targeted interventions.

One area of special worry is the possibility that genes may combine to initiate a harmful loop that exacerbates the severity of both diabetes and gangrene. As a result, the use of targeted medication therapy in early diabetic patient interventions appears to be a beneficial tactic to prevent gangrene from developing. This all-encompassing strategy includes managing the internal environmental disruptions that come with diabetes in order to support physiological homeostasis. It also emphasizes how critical it is to act quickly to halt the growth of diabetes and gangrene, thereby successfully breaking the harmful cycle.

This study exhibits several commendable strengths. Firstly, the adoption of a Mendelian Randomization (MR) design facilitates the establishment of causal inferences while mitigating the impact of confounding bias and reverse causation. Secondly, the scope of the analysis is confined to individuals of European ancestry, thereby minimizing the potential for spurious associations arising from population stratification. The substantial F-statistic further underscores the minimal likelihood of weak instrument bias.

However, certain limitations warrant acknowledgment. Primarily, it is imperative to recognize the distinctions between an MR study and a randomized controlled trial. Secondly, potential biases may emanate from non-random participant selection and population stratification, aspects not directly addressed by MR methodology. Thirdly, concerns related to horizontal pleiotropy and weak instrument bias may influence MR analysis outcomes. Additionally, the observed heterogeneity in results is primarily attributed to concealed confounding factors and insufficient sample size. Notably, the absence of evidence of pleiotropy in the heterogeneity analysis results assures the reliability of the data used for analysis, affirming its minimal impact on the final findings. Sensitivity analyses have been diligently conducted to mitigate potential biases, affirming the robustness of the study results.

## Conclusion

This study revealed a significant causal association between hyperglycemia and the risk of gangrene. Through a systematic analysis of 12 drugs and six key genes (IGF1, IL17A, ACE, FGF2, IL6 and TLR4), we have provided a new scientific perspective on gangrene prevention in diabetic patients. These findings not only shed light on the molecular mechanisms of disease occurrence, but also provide important guidance for future precision medicine. However, we recognize that further cross-population, large-scale clinical validation studies are critical to confirm the generalizability of these findings.

## Supplementary Material

Supplementary tables.

## Figures and Tables

**Table 1:** Potential drugs targeting genes with diabetes and gangrene association

Number	Drug	Gene	Type	Score	PMID
1	DUSIGITUMAB	IGF1	Inhibitor	30.91	None
2	RESATORVID	TLR4	Agonist	11.24	None
3	SUCRALFATE	FGF2	Agonist	5.89	7948825
4	SECUKINUMAB	IL17A	Antibody, Agonist	7.73	25354738
5	IXEKIZUMAB	IL17A	Antibody, Agonist	7.73	None
6	SILTUXIMAB	IL6	Inhibitor	9.89	8823310
7	OLOKIZUMAB	IL6	Inhibitor	9.89	24641941
8	CLAZAKIZUMAB	IL6	Inhibitor	7.42	None
9	CILAZAPRIL	ACE	Inhibitor	24.73	19290794
10	SPIRAPRIL	ACE	Inhibitor	8.99	11929321
11	FOSINOPRIL	ACE	Inhibitor	7.87	11030016
12	MOEXIPRIL	ACE	Inhibitor	7.87	11929321

Each drug-gene interaction ensured that the hypothetical drug had an expected effect on the condition, whose screening criteria was that the interacting score should be higher than 5. The link to the source was tracked to confirm the report and evaluate related metadata. Drugs that targeted the candidate genes through appropriate interactions were collected in the final list. *The score is the combined number of database sources and Pubmed references.
