# Supplementary material for: Repurposing Antidiabetic Drugs for Gangrene: A Mendelian Randomization and Text Mining Study
Source: Int J Med Sci. 2025 Jun 12;22(12):2896–905. doi: 10.7150/ijms.111050 (PMC12243868; doi:10.7150/ijms.111050)
Supplement: Supplementary file 1 — Supplementary tables. [file ijmsv22p2896s1.pdf]

Table 1. Information of GWAS datasets used in the MR study.

	Phenotype	PMID	Sample size	Number of SNPs	Population	Sex	Category
Expose	Type 1 diabetes ebi-a-GCST90014023	34012112	(18,942+501,638)	59,999,551	European(2021)	Males and Females	Binary
Expose	Type 2 diabetes ebi-a-GCST90018926	34594039	(38,841+451,248)	24,167,560	European(2021)	Males and Females	Binary
Expose	Blood glucose levels (ebi-a-GCST90025986)	34226706	400,458	4,218,897	European(2021)	Males and Females	NA
Outcome	Gangrene finn-b-R18_GANGRENE_NOT_ELSEW_CLASSIFIED	/	209+163,123	16,380,214	European (2021)	Males and Females	Binary

Table 2. Pleiotropy test and heterogeneity test

Exposure	Outcome	Pleiotropy test MR_egger			PRESSO		Heterogeneity test MR_egger			IVW		
		Intercept	SE	p	Global Test	p	Q	Q_df	Q_pval	Q	Q_df	Q_pval
T1DM	gangrene	-0.03879448	0.027636622	0.164316754	88.70878	0.3583333	80.76502102	79	0.423731478	82.77951583	80	0. 393645168
T2DM	gangrene	0.006593571	0.03015584	0.827192996	130.4955	0.9843333	128.9824458	165	0.982625053	129.0302536	166	0. 984825164
blood glucose level	gangrene	-0.002240348	0.022721679	0.921523163	292.5483	0.573	290.8356678	295	0.557555016	290.8453897	296	0. 573640911
